# Clinicopathologic Features, Diagnosis, and Characterization of the Immune Cell Population in Canine Choroid Plexus Tumors

**DOI:** 10.3389/fvets.2019.00224

**Published:** 2019-07-16

**Authors:** Martha F. Dalton, Justin M. Stilwell, Paula M. Krimer, Andrew D. Miller, Daniel R. Rissi

**Affiliations:** ^1^Department of Pathology and Athens Veterinary Diagnostic Laboratory, University of Georgia College of Veterinary Medicine, Athens, GA, United States; ^2^Section of Anatomic Pathology, Department of Biomedical Sciences, Cornell University College of Veterinary Medicine, Ithaca, NY, United States

**Keywords:** choroid plexus tumor, tumor microenvironment, neuropathology, brain, dog

## Abstract

The World Health Organization characterizes human choroid plexus tumor (CPT) as papilloma (CPP), atypical CPP (ACPP), and carcinoma (CPC). CPCs can disseminate via cerebrospinal fluid and be mistaken for metastatic carcinoma, creating a diagnostic challenge. Kir7.1 immunohistochemistry (IHC) is a highly reliable tool for diagnostic confirmation of CPTs and their differentiation from metastatic carcinomas in human beings and dogs. This study describes the neuropathology, Kir7.1 staining profile, and the immune cell population within the tumor microenvironment in 11 CPTs in dogs. Archived tissue sections with a diagnosis of CPT were examined and immunolabelled with Kir7.1 for diagnostic confirmation. The number of Ki67-positive neoplastic cells was calculated in 2.4 mm^2^ (equivalent to 10 FN22/40X fields), and a mean value was generated for each neoplasm. IHC for CD3, CD20, MAC387, and Iba1 was performed for immune cell characterization, and the number of stained cells for each antibody was counted in 2.4 mm^2^, generating individual cumulative values for each antibody. *T*-tests with Bonferroni correction evaluated IHC differences between tumor types, and Spearman's rank correlations evaluated relationships among IHC markers. Kir7.1 immunoreactivity was intense at the apical cell membrane in CPPs and ACPPs, and at the apical cell membrane and cytoplasm in CPCs. Ki67 immunoreactivity was detected in all cases. CD3+ and CD20+ lymphocytes trended together (*p* = 0.005) and were present within and around all CPTs. Five cases had intravascular MAC387+ monocytes. Iba1 immunoreactivity was robust within and around all tumors. Statistical differences in immune cell markers were not found among tumor types. As previously reported, Kir7.1 is a reliable antibody for the diagnosis of canine CPTs. Although immune cells were present in all cases, no significant associations were found between the type of cells and tumor diagnosis. The characterization of the immune cells within CPTs could be useful in future studies involving immunotherapy.

## Introduction

Choroid plexus tumors (CPTs) are intraventricular neoplasms that arise from the choroid plexus epithelium (1, 2). In human beings, CPTs account for <1% of all intracranial neoplasms in the overall population and about 12–35% of those occurring in the first year of life (3, 4). In veterinary medicine, CPTs occur predominantly in dogs (5–7) and are rare to unreported in other species (5–14). In dogs, CPTs account for 10% of all primary central nervous system (CNS) neoplasms (7), occurring mainly in middle-aged to older individuals (2, 6, 7, 15). Magnetic resonance imaging has been shown to be useful in the clinical diagnosis of intraventricular masses, but diagnostic confirmation of a CPT relies heavily on histology and immunohistochemistry (IHC) (6, 7, 15).

The characterization of canine CPTs is currently based on the World Health Organization (WHO) classification system for human CNS neoplasms (1). In this classification system, tumor grade is the most important prognostic factor that correlates with the overall survival time after surgery (1). However, no such correlations have been drawn in veterinary medicine, as canine patients are often subjected to euthanasia prior to surgery or any other therapeutic intervention is implemented (16). Treatment of human CPTs relies on surgery and radiation therapy, especially in the case of more invasive tumors (1). Further, tumor immunotherapy is a relatively new therapeutic approach that aims to regulate the immune response against tumor antigens and increase tumor regression (17). Immunotherapy has been utilized in many human cancers, including glial tumors (18). Little evidence is available to support or undermine specific treatment modalities in canine patients (16).

The characterization of the immune cell population within the tumor microenvironment has been conducted in a few subsets of CNS neoplasms in dogs and cats, including canine and feline meningioma, canine oligodendroglioma, and feline glioma (19–22). Such information could be useful in future immunotherapy studies involving canine and feline patients with CNS tumors. Here we describe the clinical and neuropathologic features of CPTs in 11 dogs, focusing on the characterization of the immune cell population within the tumor microenvironment.

## Materials and Methods

Cases of CPT in dogs were retrospectively identified at the Athens Veterinary Diagnostic Laboratory database between 2005 and 2018. Selected cases were reviewed, archived glass slides were examined, and replicate tissue sections were immunostained with inward rectifier potassium channel Kir7.1 for diagnostic confirmation (15). A detailed histological evaluation of all confirmed cases was performed, and tumors were characterized according to the 2016 WHO Classification of Tumors of the Central Nervous System (1). The presence of microvascular proliferation and desmoplasia was also assessed according to previously published work (23). All confirmed cases were subsequently immunostained with Ki67 (rabbit monoclonal, RTU at 60 min, Cell Marque) to assess the tumor proliferative activity; the percentage of Ki67-positive neoplastic cells was calculated in 2.4 mm^2^ (equivalent to 10 FN22/40X fields), and a mean value was generated for each neoplasm. For the characterization of the immune cell population, tissue sections were immunostained with CD3 (rabbit polyclonal, 1:1,000 dilution at 60 min, Dako), CD20 (rabbit polyclonal, 1:2,000 at 90 min, Biocare), MAC387 (mouse monoclonal, 1:500 at 60 min, Dako), and Iba1 (rabbit polyclonal, 1:8,000 at 60 min, Wako). For these immunomarkers, a cumulative value was generated after the number of stained inflammatory cells was randomly counted in 2.4 mm^2^. Immunostaining was then subjectively classified as mainly intratumoral, peritumoral, intravascular, perivascular, within or around necrotic areas, or within areas of parenchymal invasion. After confirmation of normality, a *T*-test with Bonferroni correction evaluated immunohistochemistry (IHC) differences among tumor types and Spearman's rank correlations evaluated possible relationships among immunomarkers. Linear regression was performed on immunomarkers with statistically significant relationships in the latter analysis.

## Results

Eleven cases (10 necropsies and one biopsy) met the criteria for inclusion in this study (Table 1). The original diagnoses in all cases were based on routine histology; IHC at the time of the original diagnosis was conducted in two cases and reported as inconclusive (case 5 was immunopositive for vimentin and immunonegative for pancytokeratin; case 11 was immunopositive for pancytokeratin). The age of affected dogs varied from 5 to 11 years (mean age 7.8 years) and there was no sex or breed predisposition. Clinical signs consisted of ataxia (4 cases); tetraparesis, blindness, depression, seizures, head tilt (2 cases each); and tremors, neck pain, aggression, and urinary incontinence (one case each). Magnetic resonance imaging was suggestive of a brain tumor in 4 cases. No treatment was implemented, and 10 dogs were euthanized or spontaneously died. The clinical outcome in case 11 remains unknown.

**Table 1:** Signalment, clinical signs, and clinical outcome in 11 cases of canine choroid plexus tumor.

**Case**	**Age (years)**	**Sex**	**Breed**	**Clinical signs**	**Outcome**
1	9	MN	Labrador retriever	Acute onset neurologic signs	E
2	9	F	Mixed breed	Ataxia, tremors, blindness	E
3	5	FS	Boxer	Acute onset neurologic signs	E
4	11	MN	Chow Chow	Lethargy, seizures	D
5	8	FS	Brittany spaniel	Left head tilt, ataxia	E
6	7	FS	Labrador retriever	Right head tilt	E
7	5	M	Beagle	Seizures	D
8	8	MN	Mixed breed	Neck pain, tetraparesis	E
9	9	MN	Yorkshire terrier	Aggression, blindness	E
10	6	FS	Mixed breed	Lethargy, ataxia	E
11	9	MN	German Shepherd	Ataxia, urinary incontinence	U

*MN, male neutered; E, euthanasia; F, female; FS, female spayed; D, spontaneous death; M, male; U, unknown*.

**Figure 1:**
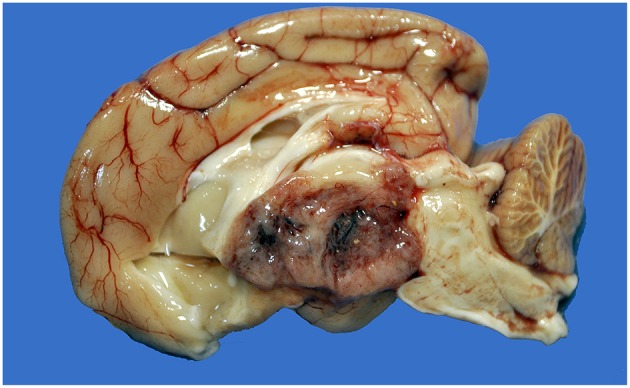
Choroid plexus carcinoma, brain, dog (case 9). A mottled tan-red neoplasm effaces the third ventricle.

Tumors were reported during necropsy examination in all cases. The most common location (Table 2) was the fourth ventricle (4 cases), followed by the lateral and third ventricles (3 cases each), and central canal of the spinal cord (2 cases). One dog (case 1) had two tumors, one in the fourth ventricle and one in the spinal canal. Neoplasms were grossly characterized as well-demarcated, red, tan, or brown masses (Figure 1). After histologic reevaluation according to the 2016 WHO classification system (1), four diagnoses (cases 4, 5, 9, and 11) were modified in relation to the original routine diagnoses (Table 2).

**Table 2:** Pathological changes, diagnosis, and immunohistochemistry in 11 cases of canine choroid plexus tumor.

**Case**	**Gross pathology and tumor location**	**Original diagnosis**	**Revised diagnosis**	**Immunohistochemistry**					
				**Kir7.1**	Ki67^*^	**CD3^**§**^**	**CD20^**§**^**	**MAC387^**§**^**	**Iba1^**§**^**
1	6 mm diameter, well-demarcated, red, soft nodules in the left cerebellopontine angle (fourth ventricle) and spinal cord (T13-L1)	CPC	CPC	+	19.8	124	60	0	322
2	5 mm diameter, well-demarcated, red, soft nodule in the left cerebellopontine angle (fourth ventricle)	CPC	CPC	+	23.4	104	52	3	480
3	15 mm diameter, well-demarcated, tan, firm nodule in the third ventricle	CPP	CPP	+	23.8	151	20	3	467
4	20 mm diameter, poorly-demarcated, brown, soft nodule in the left lateral ventricle	CPC	ACPP	+	3.2	66	57	11	363
5	20 mm diameter, well-demarcated, pale tan, soft nodule in the left cerebellopontine angle (fourth ventricle)	CPP	CPC	+	30.2	213	251	8	326
6	12 mm diameter, well-demarcated, red, soft nodule in the left cerebellopontine angle (fourth ventricle)	CPC	CPC	+	14.6	94	235	2	320
7	8 mm diameter, well-demarcated, red, soft nodule in the left lateral ventricle	CPP	CPP	+	19.6	36	6	0	326
8	Thickened choroid plexus in the lateral ventricle	CPP	CPP	+	42	41	3	0	406
9	25 mm diameter, well-demarcated, red, soft nodule in the third ventricle	Neoplasm	CPC	+	3.4	326	356	0	437
10	10 mm diameter, well-demarcated, brown, soft nodule in the third ventricle	ACPP	ACPP	+	29.4	49	12	0	612
11	Biopsy from spinal cord (L5)	CPP	ACPP	+	60.2	17	2	0	164

*CPC, choroid plexus carcinoma; CPP, choroid plexus papilloma; ACPP, atypical choroid plexus papilloma*.

* *Ki67, mean percentage of positive cells in 2.4 mm^2^*;

§ *total number of immunopositive cells in 2.4 mm^2^*.

**Figure 2:**
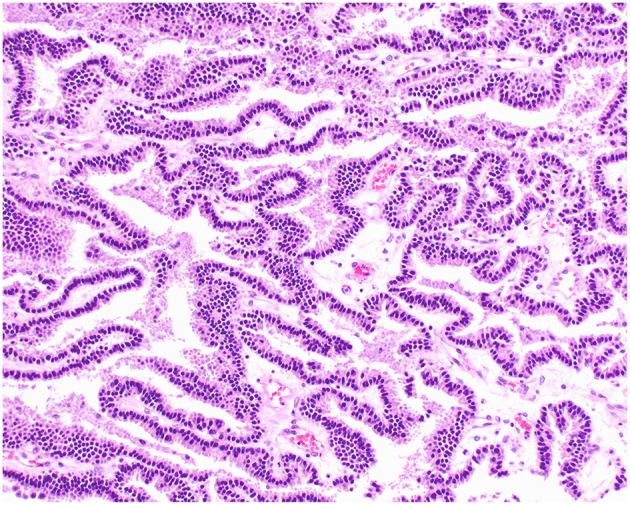
Choroid plexus papilloma, brain, dog (case 3). Neoplastic cells with low pleomorphism forming papillary projections supported by a fine fibrovascular stroma. Obj. 20x.

**Figure 3:**
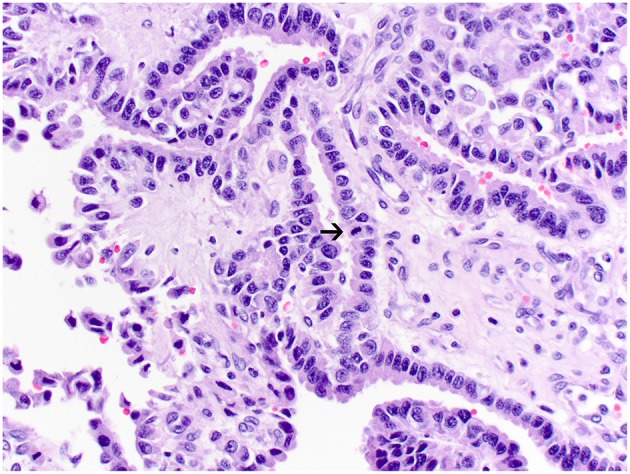
Atypical choroid plexus papilloma, brain, dog (case 4). Neoplastic cells also form papillary projections but have a mild degree of nuclear pleomorphism. Rare mitoses are present (arrow). Obj. 40x.

**Figure 4:**
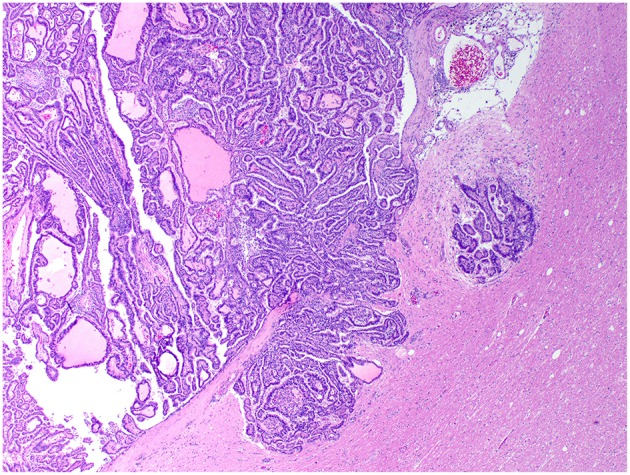
Choroid plexus carcinoma, brain, dog (case 5). A carcinoma exhibiting areas of invasion into the adjacent parenchyma, which is vacuolated and has areas of desmoplasia. Obj. 10x.

**Figure 5:**
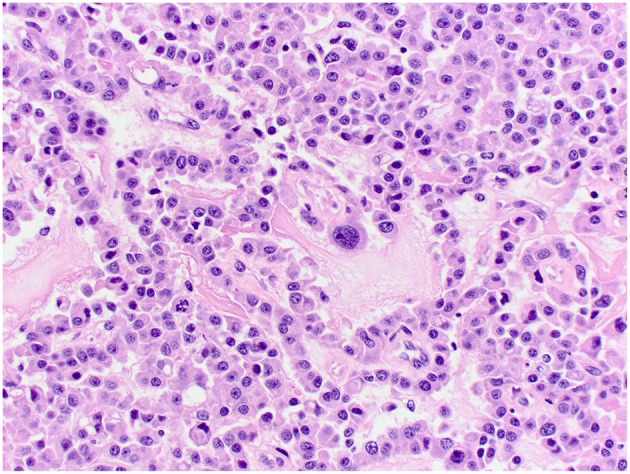
Choroid plexus carcinoma, brain, dog (case 6). Moderately pleomorphic neoplastic cells forming solid sheets and papillary areas. Obj. 40x.

Histologically, CPPs consisted of well-demarcated, papillary, moderately cellular neoplasms (Figure 2). Small areas of invasion into the adjacent neuroparenchyma were occasionally present, but no desmoplasia was detected. Single layers of neoplastic cells were arranged in papillary projections supported by scant fibrovascular fronds. Neoplastic cells were cuboidal or columnar and had eosinophilic, homogeneous or vacuolar cytoplasm and round nuclei with dense chromatin and indistinct nucleoli. There was mild cellular and nuclear pleomorphism, and mitoses were absent. There were small areas of mineralization and necrosis in all cases. ACPPs were relatively well-demarcated, papillary, and moderately cellular. Occasional areas of invasion into the adjacent neuroparenchyma without evidence of desmoplasia were present. Neoplastic cells were arranged in single or multiple layers and formed papillary fronds supported by scant fibrovascular fronds. Neoplastic cells were cuboidal or columnar and had eosinophilic, homogeneous or vacuolar cytoplasm and round nuclei with dense chromatin and indistinct nucleoli. There was moderate cellular and nuclear pleomorphism and mitotic activity was present (Figure 3), with 3 (case 4 and 10) and 5 (case 11) mitoses in 2.4 mm^2^. Multiple intratumoral areas of mineralization were present in one case. CPCs had multiple areas of invasion with vacuolation and desmoplasia of the adjacent neuroparenchyma (Figure 4). Cases 2, 6, and 9 were relatively well-demarcated but densely cellular tumors with single or multiple layers of neoplastic cells arranged in closely apposed sheets, or less often papillary projections supported by scant fibrovascular fronds (Figure 5). Neoplastic cells were cuboidal or columnar and had eosinophilic, homogeneous or vacuolar cytoplasm and round nuclei with dense chromatin and indistinct nucleoli. There was moderate cellular and nuclear pleomorphism with occasional binucleation. Mitotic activity was variable and ranged from 1 (case 1 and 2) to 7 (case 6 and 9) and up to 16 mitoses (case 5) in 2.4 mm^2^. Occasional axon spheroids were found in areas of vacuolation and desmoplasia surrounding the neoplasms, particularly in areas near the leptomeninges.

**Figure 6:**
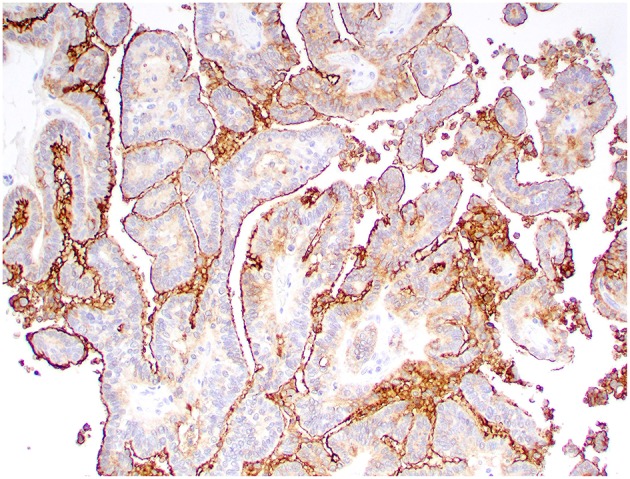
Choroid plexus papilloma, brain, dog (case 8). Intense Kir7.1 immunolabeling at the apical cell membrane. Obj. 20x.

**Figure 7:**
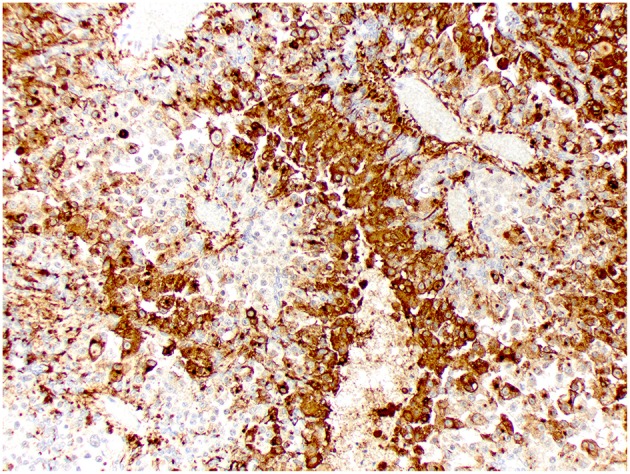
Choroid plexus carcinoma, brain, dog (case 9). Intense Kir7.1 immunolabeling at the apical cell membrane and cytoplasm. Obj. 20x.

**Figure 8:**
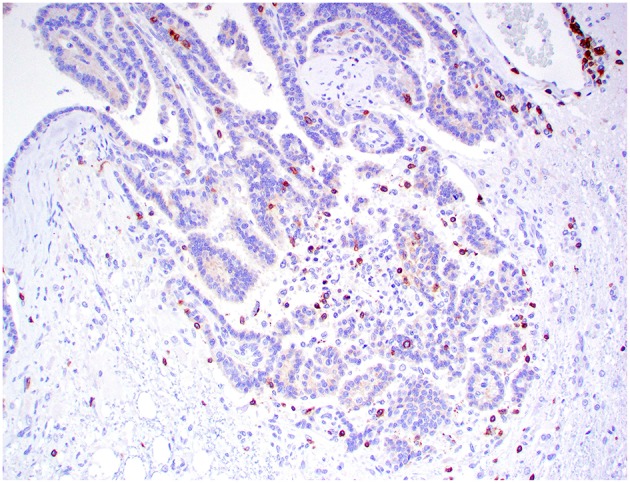
Choroid plexus carcinoma, brain, dog (case 5). CD3+ T lymphocytes are scattered throughout the tumor. Obj. 20x.

**Figure 9:**
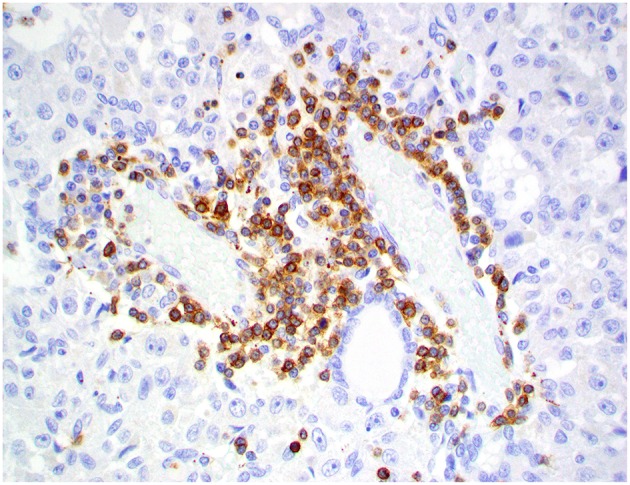
Choroid plexus carcinoma, brain, dog (case 9). CD3+ T lymphocytes are present around blood capillaries within the neoplasm. Obj. 40x.

**Figure 10:**
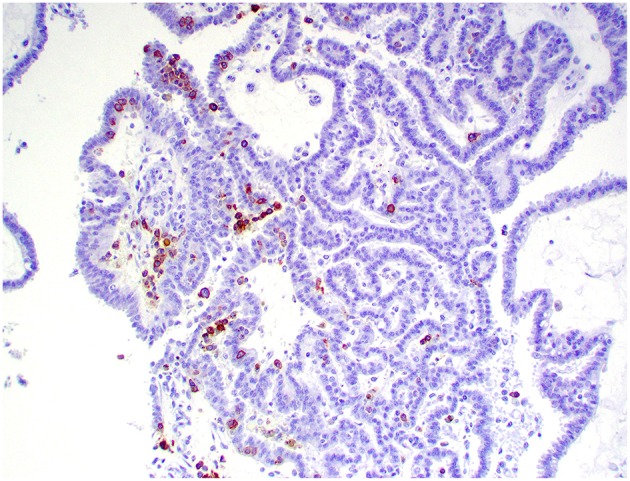
Choroid plexus carcinoma, brain, dog (case 5). CD20+ B lymphocytes are scattered throughout the tumor. Obj. 20x.

**Figure 11:**
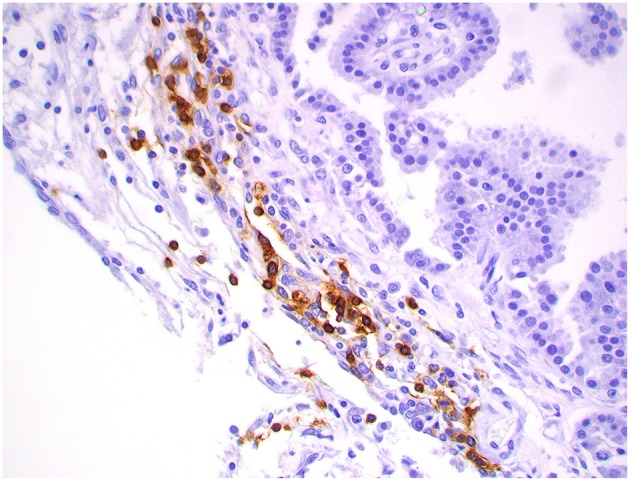
Choroid plexus carcinoma, brain, dog (case 1). CD20+ B lymphocytes are clustering around blood capillaries within the neoplasm. Obj. 40x.

**Figure 12:**
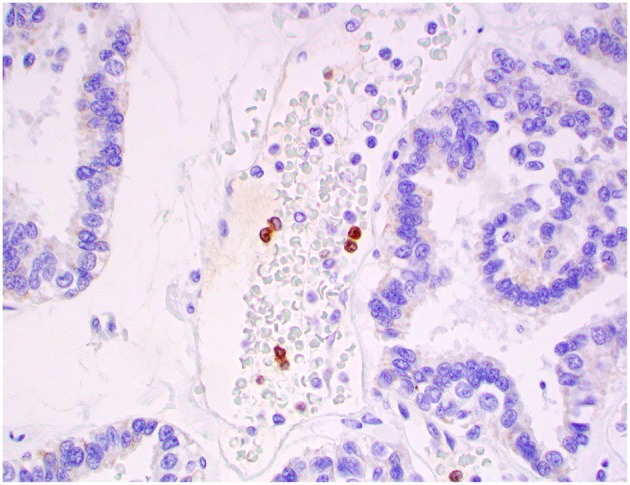
Atypical choroid plexus papilloma, brain, dog (case 4). MAC387+ monocytes occurred strictly within blood capillaries. Obj. 40x.

**Figure 13:**
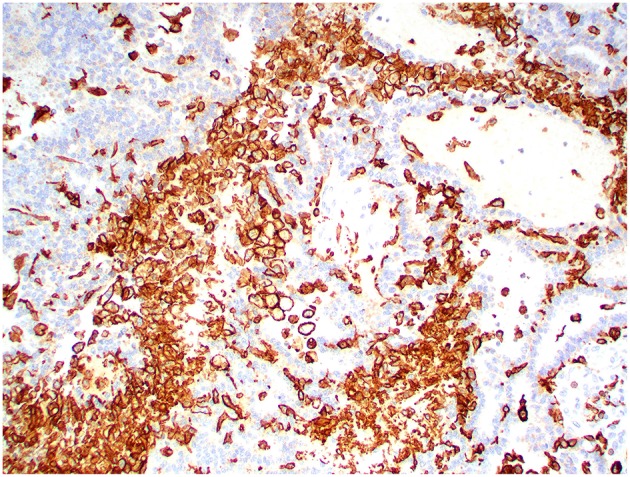
Choroid plexus carcinoma, brain, dog (case 5). Iba1 immunoreactivity was robust in all tumors and occurred within and around the tumors. Obj. 20x.

Kir7.1 immunoreactivity was detected in all cases. Immunolabeling was intense at the apical cell membrane in CPPs and ACPPs (Figure 6), and intense at the apical cell membrane and cytoplasm in CPCs (Figure 7). Ki67 immunoreactivity was detected in all cases, but significant variation was not detected among tumor diagnoses. CD3+ T lymphocytes and CD20+ B lymphocytes were present in all cases, and occurred mainly within perivascular spaces. CD3+ T lymphocytes (Figures 8, 9) were perivascularly located within tumors (6 cases) and around tumors (5 cases). CD20+ B lymphocytes (Figures 10, 11) were perivascularly located around tumors (5 cases), within and around tumors (4 cases) and strictly within tumors (2 cases). MAC387 immunoreactivity (Figure 12) occurred in 5 cases and highlighted intravascular monocytes within the tumors. Robust Iba1 immunoreactivity was observed in all cases. Iba1+ cells had long cytoplasmic processes and were diffusely interspersed with neoplastic cells and also around the tumors (Figure 13). CD3+ and CD20+ lymphocytes trended together (*p* = 0.005, r^2^ = 0.7005), but other statistical correlations among immune cell markers were not found within or between tumor types (Supplementary Figure 1).

## Discussion

As observed in the current study, CPTs are uncommon in dogs (7), representing only 0.19% of 5,680 canine necropsies submitted to our diagnostic service between 2005 and 2018. In contrast to human CPTs, which are primarily pediatric neoplasms, canine CPTs occur mainly in middle-aged to older individuals (2, 6, 7, 15), as corroborated by the current dataset.

The diagnosis of canine CPTs, as well as other CNS neoplasms, still relies heavily on tumor location, morphology, and IHC (6, 7, 15). The classification of these neoplasms is firmly rooted in outdated veterinary grading systems or systems developed for human CNS tumors (1, 24). The WHO classification system has been designed to cluster specific tumor subsets according to their morphologic and molecular features and their respective clinical outcome. These criteria cannot be entirely applied to veterinary species due to the lack of data related to post-surgical and post-treatment survival times (16). Based on the 2016 WHO grading system, the diagnosis of three of the current cases was different than the original routine diagnosis. However, it remains undetermined whether these differences would have been clinically significant, as all patients died or were euthanized after the diagnosis, as is usually the case with canine CNS tumors (16).

Unlike CPP and ACPP, CPC poses a diagnostic challenge for the pathologist, as tumors need to be differentiated from ependymal neoplasms or metastatic carcinomas (3, 15). This assessment can be especially problematic when examining biopsy samples, which typically have a limited amount of tissue available (25). IHC for cytokeratin, glial fibrillary acidic protein (GFAP), E-cadherin, N-cadherin, and beta-catenin can be useful to support a diagnosis of CPT, but these immunomarkers lack specificity and often provide inconsistent results (26–28). Here we confirm that Kir7.1 is a reliable antibody for diagnostic confirmation of choroid plexus neoplasms in dogs. As reported previously, Kir7.1 is highly specific for choroid plexus epithelium and is able to differentiate CPTs from other primary or metastatic brain tumors, including metastatic carcinomas (3, 15).

A higher proliferative activity in human CPCs tends to be associated with a less favorable post-surgical outcome when compared to CPPs (29, 30). A large population study in dogs has shown a similar trend, with a higher proliferative activity in CPCs than CPPs (23). However, similar to what we found in this group of dogs, other studies have not confirmed that finding (31, 32). While this can be related to the limitations of examining a small number of cases (31, 32), these discrepancies indicate that no conclusions should be drawn until a greater number of tumors can be assessed. Desmoplasia and microvascular proliferation have also been reported as potential indicators of malignancy in canine CPTs (23). Similarly, we found peritumoral desmoplasia within areas of invasion in 4 of 5 CPCs, in 2 of 3 ACCPs, and in none of the CPPs. Microvascular proliferation was not a prominent feature in our cases.

The use of immune therapy can be useful not only in the recruitment of immune cells to the tumor microenvironment, but also in the reinforcement of immunological memory to prevent further tumor recurrence (33). Tumor reduction and clinical improvement has been demonstrated using immunotherapy in canine brain tumors, but more comprehensive studies are needed to generate data related to the effectiveness of this treatment modality in canine patients (34–36). In human beings, CD4+ and CD8+ cytotoxic T lymphocytes are well-suited for the destruction of glioma cells due to their ability to recognize specific tumor antigens on the cell surface and have been shown to correlate with tumor inhibition and tumor regression (37). In contrast, regulatory T-cells tend to suppress this T-cell response, maintaining an immunosuppressive microenvironment that limits an effective antitumoral response to other immune cells (37). Previous evaluations of the immune cell infiltration in canine and feline CNS neoplasms have characterized the majority of infiltrating lymphocytes as CD3+ T lymphocytes, with fewer B lymphocytes (19, 22). These results are different than those in this study, in that intratumoral CD3+ and CD20+ lymphocytes trended together in equal numbers. However, this study is limited by a low number of cases overall and within each group, so these results should be interpreted cautiously and ideally replicated in future assessments of the CPT microenvironment in dogs. Further, the role of regulatory T-cells in canine cancer is still poorly understood and future studies evaluating their presence in canine CNS tumors would be useful to determine their potential role as antitumor immunity suppressors (38). Iba1 immunoreactivity was robust and observed within and around tumors in all 11 of our cases. In contrast, MAC387 immunoreactivity occurred in only 5 cases and was restricted to intravascular monocytes. It has been hypothesized that the inability of MAC387 to react with microglia and the prominent cytoplasmic ramifications present in the Iba1+ cells may suggest that the latter represents proliferating microglia rather than migrating systemic monocytes/macrophages (19, 20). Recruitment of peripheral monocytes that differentiate to tissue macrophages and microglial proliferation has been reported in both human and veterinary intracranial neoplasms (19, 21, 22, 39). Increased numbers of microglia and macrophages are associated with malignancy in human glioblastoma (39); while the exact role of these immune cells is still unknown, it is possible that they act in an inherent regulatory mechanism and prevent T lymphocyte expansion within the tumor microenvironment (40). Given the plasticity of macrophages and microglia, a definitive differentiation between these two cell populations cannot be achieved with current available immunomarkers. Further, because of sample size limitations and lack of specific studies, no conclusions can be made on the predominance and role of macrophages and microglia in canine CNS tumors.

## Author Contributions

DR: conception and design. MD, JS, PK, AM, and DR: data analysis and writing and manuscript revision.

### Conflict of Interest Statement

The authors declare that the research was conducted in the absence of any commercial or financial relationships that could be construed as a potential conflict of interest.

## Supplementary Material

Supplementary Figure 1 CD3^+^ and CD20^+^ lymphocytes trended together (*p* = 0.005, *r*^2^ = 0.7005) in the examined samples.
