# Supplementary figures and images for: Clinicopathologic Features, Diagnosis, and Characterization of the Immune Cell Population in Canine Choroid Plexus Tumors
Source: Front Vet Sci. 2019 Jul 16;6:224. doi: 10.3389/fvets.2019.00224 (PMC6646530; doi:10.3389/fvets.2019.00224)

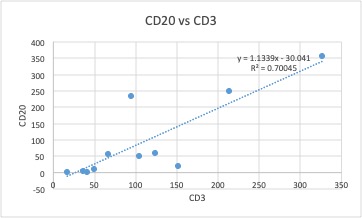

Supplement: Supplementary Figure 1 — CD3+ and CD20+ lymphocytes trended together (p = 0.005, r2 = 0.7005) in the examined samples. [file Image_1.JPEG]
